# Supplementary material for: Modeling glioblastoma heterogeneity as a dynamic network of cell states
Source: Mol Syst Biol. 2021 Sep 16;17(9):e10105. doi: 10.15252/msb.202010105 (PMC8444284; doi:10.15252/msb.202010105)
Supplement: Supplementary file 5 — Source Data for Figure 3 [file MSB-17-e10105-s001.zip › Figure3A_sourcedata/GSEA_3065/hallmarks_state3.GseaPreranked.1623416274744/HALLMARK_OXIDATIVE_PHOSPHORYLATION.html]

Details for gene set HALLMARK\_OXIDATIVE\_PHOSPHORYLATION[GSEA]

|  || Dataset | state3 |
| Phenotype | NoPhenotypeAvailable |
| Upregulated in class | na\_neg |
| GeneSet | HALLMARK\_OXIDATIVE\_PHOSPHORYLATION |
| Enrichment Score (ES) | -0.6553992 |
| Normalized Enrichment Score (NES) | -2.559873 |
| Nominal p-value | 0.0 |
| FDR q-value | 0.0 |
| FWER p-Value | 0.0 |
Table: GSEA Results Summary

  

Fig 1: Enrichment plot: HALLMARK\_OXIDATIVE\_PHOSPHORYLATION      
 Profile of the Running ES Score & Positions of GeneSet Members on the Rank Ordered List

  

| PROBE | GENE SYMBOL | GENE\_TITLE | RANK IN GENE LIST | RANK METRIC SCORE | RUNNING ES | CORE ENRICHMENT || 1 | POR |  |  | 239 | 0.269 | -0.0188 | No |
| 2 | ATP6V0B |  |  | 319 | 0.238 | -0.0199 | No |
| 3 | ATP6AP1 |  |  | 487 | 0.201 | -0.0325 | No |
| 4 | NDUFC2 |  |  | 494 | 0.199 | -0.0264 | No |
| 5 | LRPPRC |  |  | 754 | 0.163 | -0.0511 | No |
| 6 | HADHA |  |  | 1099 | 0.130 | -0.0868 | No |
| 7 | ATP1B1 |  |  | 1102 | 0.129 | -0.0827 | No |
| 8 | NDUFA9 |  |  | 1400 | 0.108 | -0.1137 | No |
| 9 | AFG3L2 |  |  | 1452 | 0.105 | -0.1160 | No |
| 10 | SDHA |  |  | 1487 | 0.104 | -0.1165 | No |
| 11 | NNT |  |  | 1657 | 0.094 | -0.1330 | No |
| 12 | OGDH |  |  | 1708 | 0.091 | -0.1357 | No |
| 13 | FH |  |  | 1813 | 0.086 | -0.1449 | No |
| 14 | RHOT2 |  |  | 1876 | 0.082 | -0.1494 | No |
| 15 | MRPS22 |  |  | 2205 | 0.068 | -0.1853 | No |
| 16 | ATP6V1H |  |  | 2249 | 0.066 | -0.1881 | No |
| 17 | IDH1 |  |  | 2376 | 0.062 | -0.2007 | No |
| 18 | HSPA9 |  |  | 2404 | 0.060 | -0.2018 | No |
| 19 | ABCB7 |  |  | 2552 | 0.055 | -0.2171 | No |
| 20 | DLD |  |  | 2811 | 0.047 | -0.2456 | No |
| 21 | OPA1 |  |  | 2868 | 0.045 | -0.2506 | No |
| 22 | NDUFB8 |  |  | 2871 | 0.045 | -0.2493 | No |
| 23 | COX15 |  |  | 2959 | 0.042 | -0.2580 | No |
| 24 | LDHB |  |  | 3031 | 0.040 | -0.2649 | No |
| 25 | IMMT |  |  | 3080 | 0.039 | -0.2692 | No |
| 26 | PDHA1 |  |  | 3126 | 0.038 | -0.2732 | No |
| 27 | AIFM1 |  |  | 3137 | 0.038 | -0.2731 | No |
| 28 | HADHB |  |  | 3282 | 0.034 | -0.2887 | No |
| 29 | NDUFS2 |  |  | 3331 | 0.032 | -0.2932 | No |
| 30 | NDUFS1 |  |  | 3461 | 0.029 | -0.3073 | No |
| 31 | ACADVL |  |  | 3600 | 0.026 | -0.3225 | No |
| 32 | SUCLA2 |  |  | 3648 | 0.024 | -0.3272 | No |
| 33 | NDUFA4 |  |  | 3699 | 0.023 | -0.3323 | No |
| 34 | MAOB |  |  | 3748 | 0.021 | -0.3371 | No |
| 35 | PDHX |  |  | 3853 | 0.019 | -0.3486 | No |
| 36 | MRPS30 |  |  | 4251 | 0.008 | -0.3947 | No |
| 37 | OXA1L |  |  | 4372 | 0.005 | -0.4086 | No |
| 38 | GOT2 |  |  | 4434 | 0.003 | -0.4156 | No |
| 39 | MDH1 |  |  | 4655 | -0.003 | -0.4412 | No |
| 40 | PMPCA |  |  | 4714 | -0.004 | -0.4478 | No |
| 41 | MRPL35 |  |  | 4911 | -0.009 | -0.4704 | No |
| 42 | DLAT |  |  | 5005 | -0.011 | -0.4809 | No |
| 43 | RHOT1 |  |  | 5045 | -0.012 | -0.4850 | No |
| 44 | NDUFS7 |  |  | 5079 | -0.013 | -0.4884 | No |
| 45 | UQCRC2 |  |  | 5115 | -0.014 | -0.4920 | No |
| 46 | OAT |  |  | 5237 | -0.017 | -0.5056 | No |
| 47 | MGST3 |  |  | 5395 | -0.021 | -0.5232 | No |
| 48 | ATP6V1C1 |  |  | 5422 | -0.021 | -0.5255 | No |
| 49 | SDHD |  |  | 5493 | -0.024 | -0.5328 | No |
| 50 | HCCS |  |  | 5576 | -0.026 | -0.5415 | No |
| 51 | SUPV3L1 |  |  | 5585 | -0.027 | -0.5415 | No |
| 52 | GPI |  |  | 5670 | -0.029 | -0.5504 | No |
| 53 | VDAC1 |  |  | 5671 | -0.029 | -0.5494 | No |
| 54 | GLUD1 |  |  | 5727 | -0.031 | -0.5548 | No |
| 55 | MFN2 |  |  | 5931 | -0.037 | -0.5772 | No |
| 56 | TOMM70 |  |  | 5941 | -0.037 | -0.5770 | No |
| 57 | ACO2 |  |  | 5970 | -0.038 | -0.5789 | No |
| 58 | BDH2 |  |  | 5983 | -0.039 | -0.5790 | No |
| 59 | CS |  |  | 5985 | -0.039 | -0.5778 | No |
| 60 | IDH3G |  |  | 6007 | -0.040 | -0.5789 | No |
| 61 | ACAA1 |  |  | 6069 | -0.042 | -0.5846 | No |
| 62 | VDAC3 |  |  | 6079 | -0.042 | -0.5842 | No |
| 63 | GRPEL1 |  |  | 6088 | -0.042 | -0.5837 | No |
| 64 | ALDH6A1 |  |  | 6109 | -0.043 | -0.5846 | No |
| 65 | UQCRC1 |  |  | 6149 | -0.045 | -0.5876 | No |
| 66 | TIMM9 |  |  | 6180 | -0.046 | -0.5896 | No |
| 67 | COX11 |  |  | 6187 | -0.046 | -0.5887 | No |
| 68 | ACADM |  |  | 6280 | -0.049 | -0.5978 | No |
| 69 | ATP5MC3 |  |  | 6363 | -0.051 | -0.6056 | No |
| 70 | FXN |  |  | 6459 | -0.055 | -0.6148 | No |
| 71 | NDUFA7 |  |  | 6493 | -0.056 | -0.6167 | No |
| 72 | ALAS1 |  |  | 6560 | -0.059 | -0.6224 | No |
| 73 | CASP7 |  |  | 6580 | -0.060 | -0.6226 | No |
| 74 | NDUFB6 |  |  | 6609 | -0.061 | -0.6238 | No |
| 75 | HSD17B10 |  |  | 6762 | -0.069 | -0.6392 | No |
| 76 | ACAT1 |  |  | 6798 | -0.071 | -0.6409 | No |
| 77 | SDHC |  |  | 6808 | -0.071 | -0.6395 | No |
| 78 | NDUFS3 |  |  | 6821 | -0.072 | -0.6384 | No |
| 79 | ATP5F1A |  |  | 6828 | -0.072 | -0.6367 | No |
| 80 | TIMM17A |  |  | 6832 | -0.072 | -0.6346 | No |
| 81 | NDUFS4 |  |  | 6944 | -0.078 | -0.6449 | No |
| 82 | DECR1 |  |  | 6981 | -0.079 | -0.6464 | No |
| 83 | ATP6V1D |  |  | 6994 | -0.080 | -0.6450 | No |
| 84 | SUCLG1 |  |  | 7007 | -0.082 | -0.6437 | No |
| 85 | NDUFB5 |  |  | 7052 | -0.084 | -0.6459 | No |
| 86 | PHYH |  |  | 7066 | -0.085 | -0.6446 | No |
| 87 | IDH2 |  |  | 7068 | -0.085 | -0.6418 | No |
| 88 | ETFB |  |  | 7090 | -0.086 | -0.6413 | No |
| 89 | IDH3B |  |  | 7099 | -0.087 | -0.6393 | No |
| 90 | ATP6V0E1 |  |  | 7106 | -0.088 | -0.6370 | No |
| 91 | ETFA |  |  | 7125 | -0.089 | -0.6361 | No |
| 92 | ATP5PB |  |  | 7149 | -0.090 | -0.6357 | No |
| 93 | NQO2 |  |  | 7218 | -0.095 | -0.6404 | No |
| 94 | TIMM50 |  |  | 7229 | -0.095 | -0.6383 | No |
| 95 | ATP5F1B |  |  | 7276 | -0.099 | -0.6403 | No |
| 96 | IDH3A |  |  | 7286 | -0.099 | -0.6380 | No |
| 97 | TIMM10 |  |  | 7349 | -0.104 | -0.6417 | No |
| 98 | MTX2 |  |  | 7370 | -0.106 | -0.6404 | No |
| 99 | PDHB |  |  | 7438 | -0.112 | -0.6444 | No |
| 100 | HTRA2 |  |  | 7533 | -0.121 | -0.6513 | Yes |
| 101 | SLC25A3 |  |  | 7543 | -0.122 | -0.6482 | Yes |
| 102 | ACAA2 |  |  | 7550 | -0.122 | -0.6447 | Yes |
| 103 | SDHB |  |  | 7565 | -0.123 | -0.6422 | Yes |
| 104 | FDX1 |  |  | 7571 | -0.125 | -0.6385 | Yes |
| 105 | SLC25A5 |  |  | 7582 | -0.126 | -0.6354 | Yes |
| 106 | VDAC2 |  |  | 7645 | -0.132 | -0.6382 | Yes |
| 107 | PRDX3 |  |  | 7649 | -0.132 | -0.6340 | Yes |
| 108 | ECH1 |  |  | 7658 | -0.134 | -0.6304 | Yes |
| 109 | MRPL11 |  |  | 7661 | -0.135 | -0.6260 | Yes |
| 110 | ATP5F1C |  |  | 7677 | -0.136 | -0.6231 | Yes |
| 111 | PDP1 |  |  | 7720 | -0.142 | -0.6232 | Yes |
| 112 | ATP5MC1 |  |  | 7743 | -0.145 | -0.6208 | Yes |
| 113 | ECI1 |  |  | 7748 | -0.146 | -0.6164 | Yes |
| 114 | MRPL15 |  |  | 7750 | -0.146 | -0.6115 | Yes |
| 115 | CYB5R3 |  |  | 7770 | -0.148 | -0.6087 | Yes |
| 116 | CYC1 |  |  | 7816 | -0.154 | -0.6087 | Yes |
| 117 | CYB5A |  |  | 7854 | -0.159 | -0.6076 | Yes |
| 118 | NDUFAB1 |  |  | 7858 | -0.160 | -0.6025 | Yes |
| 119 | COX4I1 |  |  | 7893 | -0.165 | -0.6009 | Yes |
| 120 | SURF1 |  |  | 7911 | -0.169 | -0.5971 | Yes |
| 121 | NDUFV1 |  |  | 7913 | -0.169 | -0.5915 | Yes |
| 122 | NDUFA5 |  |  | 7986 | -0.183 | -0.5937 | Yes |
| 123 | MRPS15 |  |  | 8012 | -0.187 | -0.5902 | Yes |
| 124 | TOMM22 |  |  | 8023 | -0.189 | -0.5850 | Yes |
| 125 | NDUFA1 |  |  | 8052 | -0.194 | -0.5816 | Yes |
| 126 | MRPS12 |  |  | 8054 | -0.194 | -0.5751 | Yes |
| 127 | MRPS11 |  |  | 8097 | -0.203 | -0.5731 | Yes |
| 128 | NDUFA3 |  |  | 8101 | -0.204 | -0.5665 | Yes |
| 129 | PHB2 |  |  | 8125 | -0.210 | -0.5621 | Yes |
| 130 | MPC1 |  |  | 8135 | -0.213 | -0.5559 | Yes |
| 131 | UQCRB |  |  | 8141 | -0.214 | -0.5492 | Yes |
| 132 | ATP6V1E1 |  |  | 8188 | -0.225 | -0.5469 | Yes |
| 133 | SLC25A11 |  |  | 8194 | -0.226 | -0.5398 | Yes |
| 134 | NDUFA8 |  |  | 8226 | -0.234 | -0.5355 | Yes |
| 135 | NDUFB1 |  |  | 8241 | -0.238 | -0.5290 | Yes |
| 136 | MDH2 |  |  | 8251 | -0.242 | -0.5218 | Yes |
| 137 | POLR2F |  |  | 8262 | -0.244 | -0.5147 | Yes |
| 138 | NDUFB7 |  |  | 8276 | -0.248 | -0.5078 | Yes |
| 139 | COX7A2L |  |  | 8277 | -0.248 | -0.4993 | Yes |
| 140 | NDUFV2 |  |  | 8278 | -0.250 | -0.4908 | Yes |
| 141 | ATP6V1G1 |  |  | 8284 | -0.254 | -0.4827 | Yes |
| 142 | SLC25A4 |  |  | 8291 | -0.256 | -0.4747 | Yes |
| 143 | UQCRFS1 |  |  | 8310 | -0.260 | -0.4680 | Yes |
| 144 | ECHS1 |  |  | 8325 | -0.264 | -0.4606 | Yes |
| 145 | ISCA1 |  |  | 8330 | -0.265 | -0.4521 | Yes |
| 146 | COX6C |  |  | 8341 | -0.270 | -0.4441 | Yes |
| 147 | NDUFS8 |  |  | 8354 | -0.275 | -0.4361 | Yes |
| 148 | NDUFB3 |  |  | 8362 | -0.278 | -0.4274 | Yes |
| 149 | MRPL34 |  |  | 8365 | -0.279 | -0.4182 | Yes |
| 150 | ATP5PD |  |  | 8394 | -0.291 | -0.4115 | Yes |
| 151 | ATP5F1D |  |  | 8400 | -0.296 | -0.4020 | Yes |
| 152 | CYCS |  |  | 8405 | -0.297 | -0.3924 | Yes |
| 153 | NDUFA6 |  |  | 8407 | -0.298 | -0.3823 | Yes |
| 154 | BAX |  |  | 8408 | -0.299 | -0.3722 | Yes |
| 155 | NDUFC1 |  |  | 8422 | -0.302 | -0.3634 | Yes |
| 156 | NDUFA2 |  |  | 8437 | -0.309 | -0.3545 | Yes |
| 157 | TIMM13 |  |  | 8438 | -0.309 | -0.3440 | Yes |
| 158 | ATP5ME |  |  | 8445 | -0.312 | -0.3341 | Yes |
| 159 | COX7B |  |  | 8447 | -0.314 | -0.3235 | Yes |
| 160 | COX5B |  |  | 8448 | -0.314 | -0.3128 | Yes |
| 161 | TIMM8B |  |  | 8451 | -0.315 | -0.3023 | Yes |
| 162 | COX17 |  |  | 8453 | -0.316 | -0.2917 | Yes |
| 163 | COX5A |  |  | 8481 | -0.331 | -0.2836 | Yes |
| 164 | COX7A2 |  |  | 8507 | -0.342 | -0.2749 | Yes |
| 165 | ATP5PF |  |  | 8513 | -0.344 | -0.2637 | Yes |
| 166 | ISCU |  |  | 8531 | -0.352 | -0.2537 | Yes |
| 167 | COX7C |  |  | 8546 | -0.362 | -0.2430 | Yes |
| 168 | ATP5MF |  |  | 8547 | -0.362 | -0.2307 | Yes |
| 169 | NDUFB4 |  |  | 8560 | -0.372 | -0.2194 | Yes |
| 170 | ATP5MG |  |  | 8574 | -0.384 | -0.2079 | Yes |
| 171 | SLC25A6 |  |  | 8580 | -0.386 | -0.1953 | Yes |
| 172 | ATP5PO |  |  | 8581 | -0.386 | -0.1822 | Yes |
| 173 | NDUFB2 |  |  | 8585 | -0.391 | -0.1692 | Yes |
| 174 | ATP6V1F |  |  | 8587 | -0.392 | -0.1560 | Yes |
| 175 | ATP5MC2 |  |  | 8605 | -0.405 | -0.1442 | Yes |
| 176 | UQCR10 |  |  | 8608 | -0.410 | -0.1304 | Yes |
| 177 | COX8A |  |  | 8619 | -0.422 | -0.1172 | Yes |
| 178 | COX6B1 |  |  | 8631 | -0.435 | -0.1037 | Yes |
| 179 | NDUFS6 |  |  | 8651 | -0.456 | -0.0904 | Yes |
| 180 | UQCR11 |  |  | 8686 | -0.503 | -0.0772 | Yes |
| 181 | UQCRH |  |  | 8704 | -0.524 | -0.0614 | Yes |
| 182 | UQCRQ |  |  | 8726 | -0.602 | -0.0433 | Yes |
| 183 | ATP5F1E |  |  | 8737 | -0.662 | -0.0220 | Yes |
| 184 | GPX4 |  |  | 8742 | -0.690 | 0.0011 | Yes |
Table: GSEA details [plain text format]

  

Fig 2: HALLMARK\_OXIDATIVE\_PHOSPHORYLATION: Random ES distribution      
 Gene set null distribution of ES for **HALLMARK\_OXIDATIVE\_PHOSPHORYLATION**
